# **Beyond mobility: A prospective study on diet and metabolism in hereditary spastic paraplegia**

**DOI:** 10.1007/s11011-026-01815-x

**Published:** 2026-03-03

**Authors:** Christina Erhardt, Imke T. Spatz, Hans J. Herrmann, Zacharias Kohl, Yurdagül Zopf, Heiko Gassner, Jürgen Winkler, Martin Regensburger

**Affiliations:** 1 Department of Molecular Neurology, Friedrich-Alexander-Universität Erlangen- Nürnberg, Erlangen, Germany; 2 Hector-Center for Nutrition, Exercise and Sports, Department of Medicine 1, Friedrich-Alexander-Universität Erlangen-Nürnberg, Erlangen, Germany; 3 Department of Neurology, University Hospital Regensburg, Regensburg, Germany; 4 Fraunhofer Institute for Integrated Circuits IIS, Erlangen, Germany; 5 Deutsches Zentrum Immuntherapie (DZI), Erlangen, Germany; 6 Center for Rare Diseases Erlangen (ZSEER), University Hospital Erlangen, Erlangen, Germany Schwabachanlage 6, 91054

**Keywords:** Hereditary spastic paraplegia, Nutrition, Obesity, Body composition, Physical activity, Hypothalamic atrophy

## Abstract

Metabolism plays an important role in neurodegenerative diseases. Hereditary spastic paraplegias (HSP) are a heterogeneous group of rare genetic neurodegenerative disorders, commonly characterized by the clinical syndrome of progressive lower limb spasticity and mobility loss. Obesity has been linked to distinct genotypes, but the role of metabolism and nutrition in HSP remains unclear.We aimed to To evaluate metabolism and nutrition in specific HSP genotypes and to assess the impact of nutritional counseling on disease progression and body composition. In this prospective explorative pilot study, we assessed the neurological, metabolic and nutritional status of patients with HSP at baseline and one year after nutritional counseling. A total of 36 patients with genetically confirmed SPG4-, SPG7- and SPG11-associated HSP were recruited. BMI in SPG4 and SPG7 was comparable to healthy population data, whereas SPG11 showed significantly higher BMI (+ 22.9%, p < 0.05) with a considerable interindividual variability. Across all genotypes, disease severity according to the Spastic Paraplegia Rating Scale correlated negatively with leg muscle mass (ρ = -0.39, p < 0.05), protein (ρ = -0.35, p < 0.05) and fiber intake (ρ = -0.41, p < 0.05). After one year, there was a significant loss of relative muscle mass (-7.2%, p < 0.001). Progressive loss of muscle mass in HSP asks for an effective nutritional intervention combined with exercise in order to influence disease progression in HSP. The SPG11-associated obese phenotype may evolve with disease progression due to multifactorial metabolic changes, beyond reduced mobility.

**Supplementary Information:**

The online version contains supplementary material available at 10.1007/s11011-026-01815-x.

## Introduction

Metabolic alterations in neurodegenerative diseases are of growing interest. In amyotrophic lateral sclerosis (ALS), hypothalamic atrophy is associated with a catabolic state (Bouteloup et al. 2009; Fayemendy et al. 2021) and low body mass index (BMI) (Liu et al. 2022), which has been associated with reduced survival rates (Chang et al. 2023; Dupuis et al. 2011; Mariosa et al. 2017; Peter et al. 2017). Increased resting energy expenditure was shown in ALS, along with decreased appetite irrespective of dysphagia (Desport et al. 2000).

Acquired hypothalamic obesity describes an effect contrasting to ALS, where hypothalamic atrophy is related to obesity, hyperphagia and reduced energy expenditure in individuals with hypothalamic damage of different etiology (neoplasia, trauma, iatrogenic) (Orban et al. 2007; Argente et al. 2025). The vital role of the hypothalamus in regulating metabolic homeostasis (Brüning and Fenselau 2023; Cornejo et al. 2016; Fong et al. 2023) suggests a direct link between hypothalamic atrophy and specific changes in metabolism also for neurodegenerative diseases.

Biallelic pathogenic variants in the gene *SPG11* (also designated *ALS5*) lead to a phenotypic spectrum of juvenile amyotrophic lateral sclerosis type 5 and complicated hereditary spastic paraplegia, involving both upper and lower motor neurons, thus causing a rapidly progressive spastic paraparesis and/ or peripheral motor neuropathy (Hehr et al. 2007; Orlacchio et al. 2010; Schüle et al. 2016; Winner et al. 2004).

Hereditary spastic paraplegia (HSP) is a heterogeneous group of rare, inherited neurodegenerative disorders, typically characterized by progressive, symmetrical leg spasticity, leading to walking disability (Blackstone 2018; Fink 2013). A length-dependent degeneration of corticospinal tract neurons is the common structural-functional mechanism in HSP (Fink 2013; List et al. 2019). Based on the specific gene locus, HSP-related genotypes are classified into “SPGs” (spastic paraplegia genes) (Fink 2013).

There is evidence of relative hypothalamic atrophy and elevated BMI in SPG11-HSP patients. These findings were initially observed in a Brazilian cohort compared to individuals with Friedreich’s ataxia (Cardozo-Hernández et al. 2020). In a past study on neurometabolic function in SPG11-related HSP, our research group has demonstrated significantly higher BMI, body water and fat tissue with reduced lean tissue in patients as compared to healthy controls. Moreover, we confirmed hypothalamic atrophy in these patients using MRI volumetry (Regensburger et al. 2022a, b).

However, comprehensive data on metabolism, nutrition, as well as detailed analyses of body composition in HSP (including the most frequent SPG4 and SPG7 subtypes (Fink 2023; Hazan et al. 1999; Schüle et al. 2016) remain limited. The aim of this pilot study was to (i) characterize metabolic parameters in a representative HSP cohort, including analyses of body composition, dietary patterns, physical activity and nutrition, and to (ii) assess the impact of nutritional counseling on body composition, disease severity, and disease progression. These data offer valuable insights into the role of environmental and metabolic factors in HSP and may inform the development of potential interventional strategies aimed at modifying metabolism and thereby disease progression.

## Methods

### Inclusion and exclusion criteria

The study protocol was approved by the Institutional Review Board (Ethics Committee of Friedrich-Alexander-Universität Erlangen-Nürnberg, Erlangen, Germany, no. 347_17B, issued 26th Jan 2018). All participants provided written informed consent before enrollment. Inclusion criteria were age ≥ 18 years and a clinically and genetically confirmed diagnosis of HSP. Participants were recruited from the movement disorders outpatient unit at the University Hospital Erlangen, Germany. Only those participants who were able to stand and walk at least 10 m with or without walking aid were included. Exclusion criteria included concomitant diagnoses affecting lipid or cortisol metabolism, and the presence of eating disorders. The target recruitment was 15 participants with complicated HSP and 30 with pure HSP, including at least 15 individuals with SPG4.

### Study design

**Fig. 1:**
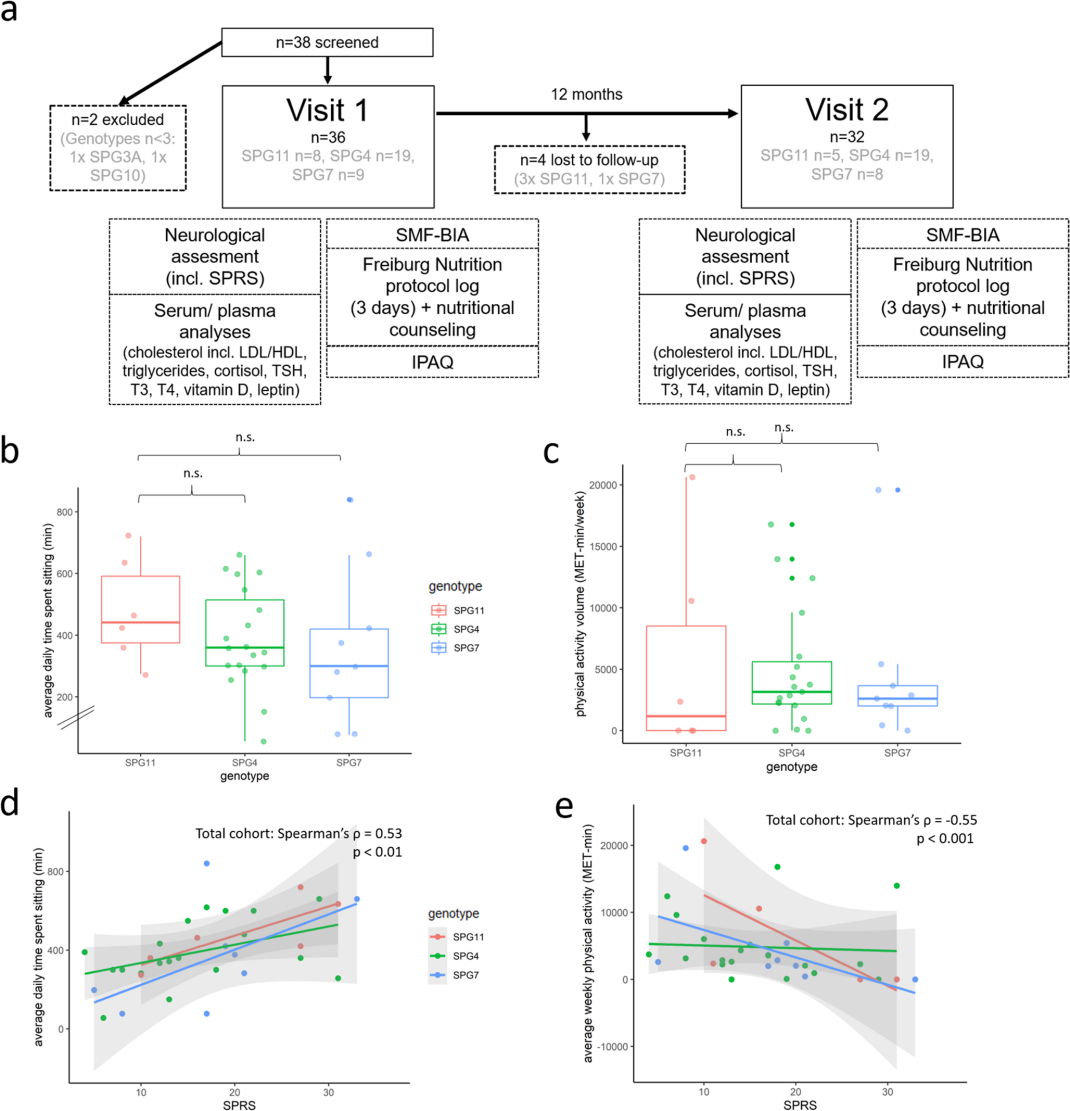
Study design and baseline physical activity. (**a**) Prospective, interventional longitudinal single-center pilot study with assessment of neurological, metabolic and nutritional status in one-year follow-up. (**b**) Average time spent sitting (minutes per day) per genotype. (**c**) Volume of physical activity (MET-min = MET x time in minutes) per genotype. (**d**) SPRS correlates with average time spent sitting inthe overall cohort (Spearman’s ρ = -0.53, p < 0.01). (**e**) SPRS correlates with average physical activity (MET-min/ week) in the overall cohort (Spearman’s ρ = -0.55, p < 0.001). *IPAQ* International Physical Activity Questionnaire, *SPRS* Spastic Paraplegia Rating Scale, *LDL* low density lipoprotein, *HDL* high density lipoprotein, *TSH* thyroidea stimulating hormone, *T3* triiodothyronine, *T4* thyroxin, *SMF-BIA* segmental multi frequency bioimpedance analysis, *MET* metabolic equivalent of task. *n.s.* not significant according to Kruskall-Wallis-Test followed by Dunn’s post-hoc test. Colors represent different genotypes

We conducted a prospective, longitudinal, interventional, single-center study. Baseline and follow-up visits (12 months later) were performed between February 2018 and January 2020 (Fig. 1a). At both timepoints, all participants completed the International Physical Activity Questionnaire (IPAQ, long form) as a patient-reported measure of daily physical activity. Data were analyzed according to the “Guidelines for Data Processing and Analysis of the International Physical Activity Questionnaire (IPAQ)” (Craig et al. 2003). To account for the duration and intensity of physical activity, average intensity of physical activity given in metabolic equivalent of task (MET) was multiplied by the time spent with physical activity in minutes. This yields a continuous measure of the volume of activity, counted in MET-min.

Three days prior to the baseline and follow-up visits, each participant completed a paper-pencil nutrition survey (recording food intake over 72 h (Trembley et al. 1983), “Freiburger Ernährungsprotokoll” by Nutri-Science GmbH, Freiburg, Germany). From these protocols, average daily energy value and macronutrients (fat, protein, carbohydrates, dietary fiber, alcohol) were calculated using the software Prodi^®^6 expert (Nutri-Science GmbH, Freiburg).

Participants were asked to avoid physical strain in the morning before the examination. Transportation to the study center was assisted. To assess metabolic and endocrinological parameters, serum samples were collected between 8:00 AM and 11:00 AM, after a minimum 8-hour fasting period. Samples were analyzed by the central laboratory of the University Hospital Erlangen for the following parameters: triglycerides, cholesterol (including HDL and LDL), thyroid stimulating hormone (TSH), triiodothyronine (T3), thyroxine (T4), cortisol, adrenocorticotropin (ACTH), leptin, and cholecalciferol (vitamin D3). Additional serum and plasma samples, including supernatants, were stored at -80 °C for future analysis.

At each visit, we assessed the neurological status, concomitant medications and dependency on walking aids. We also measured weight, height and body mass index (BMI). Disease severity was rated according to the Spastic Paraplegia Rating Scale (SPRS) (Schüle et al. 2006).

Body composition was assessed via bioelectrical impedance analysis at each visit, using the segmental multifrequency bioimpedance analysis (SMF-BIA) device “seca mBCA 515” (seca GmbH & Co. KG, Hamburg, Germany). This device measures resistance and reactance through low-intensity alternating current while the participant is standing on a platform with integrated scale holding a handrail providing postural support, with four pairs of integrated electrodes. Measurements were taken according to the manufacturer’s instructions, as previously described (Bosy-Westphal et al. 2013). An algorithm provided by the manufacturer used these measurements to calculate fat mass, fat-free mass, skeletal muscle mass, visceral fat, total body water, and extracellular water.

The intervention consisted of one session of nutritional counseling, provided by a professional dietician (Hector-Center for Nutrition, Exercise and Sports, Department of Gastroenterology, Pneumology and Endocrinology, University Hospital Erlangen). The dietician reviewed the participants’ eating habits based on their nutrition protocol and provided individualized recommendations for whole-food nutrition, in line with the current guidelines given by the German Nutrition Society (DGE). Recommendations were based on 10 key principles: (1) A diverse diet primarily consisting of plant-based foods. (2) At least three servings of vegetables and two servings of fruit daily. (3) Preference for whole-grain cereals. (4) Daily consumption of dairy products, with fish 1–2 times per week and a maximum of 300–600 g of meat per week. (5) Preference for vegetable oils. (6) Low intake of sugar and salt by avoiding added sugar and salty dishes. (7) Consumption of 1.5 L of fluid per day, preferably water or low-calorie beverages, avoiding sugary drinks. (8) Gentle food preparation methods (e.g., cooking for the minimum necessary time, using little water and fat, avoiding charring). (9) Eating slowly and mindfully. (10) Engaging in 30–60 min of moderate physical activity daily.

### Statistical analysis and illustrations

Genotypes with fewer than three participants were excluded from the final cohort. Participants taking levothyroxine were excluded from the analysis of thyroid function parameters. We used a pairwise deletion in case of missing data points. Statistical analysis as well as illustrations creation was performed using R Studio (Version 4.3.0). Kruskal-Wallis tests, followed by Dunn’s post-hoc tests, were used to compare genotypes at baseline. To assess differences between two groups at baseline (e.g. sex-specific differences, comparison of normal weight vs. obese SPG11), the non-parametric Wilcoxon test for independent samples was applied. For the analysis of longitudinal changes, the non-parametric Wilcoxon test for matched pairs was used, excluding drop-outs from the baseline data. A p-value of < 0.05 was considered statistically significant for all analyses. There was no correction for multiple testing between different analyses.

The present study focuses on body composition, nutrition, and metabolic analyses. A subpopulation of this study has been characterized on gait performance and levels of NfL (Krumm et al. 2024; Regensburger et al. 2022a, b).

## Results

### Baseline characteristics

We recruited 38 patients with clinically and genetically confirmed HSP. All participants were German and of Caucasian ethnicity. The baseline characteristics are summarized in Table 1 (for further information see supplementary table T1). The most frequent genotypes were SPG4, SPG7, and SPG11. Two patients (SPG3A and SPG10) were excluded since their genotype was underrepresented (*n* < 3). Hence, the final cohort included 36 patients from 34 families. The majority of patients was female (61.1%). None of the participants reported dysphagia.

**Table 1:** Baseline characteristics for the total cohort and specific genotypes (Mean +/- SD, both sexes) *SPRS* Spastic Paraplegia Rating Scale

*n*	Total cohort	SPG11	SPG4	SPG7
	36	8 (22.2%)	19 (52.8%)	9 (25.0%)
female: male	22:14	4:4	12:7	6:3
length of follow-up (months)	12.3 ± 1.5	12.6 ± 1.8	12.6 ± 1.3	11.5 ± 1.4
n of follow-up	32	5	19	8
age (years)	48.3 ± 13.8	25.9 ± 7.6	54.4 ± 6.8	55.3 ± 6.1
disease duration (years)	15.6 ± 11.3	16.3 ± 8.3	16.9 ± 12.4	12.4 ± 10.5
SPRS (total sum score, points)	16.6 ± 7.7	18.2 ± 7.5	15.7 ± 7.5	17.6 ± 8.0

SPG11 showed a significantly younger mean age (± SD) of 25.9 ± 7.6 compared to SPG4 and SPG7 (SPG4: 54.4 ± 6.8; SPG7: 55.3 ± 4.1; *p* < 0.001). The disease duration of the overall cohort averaged 15.6 (± 11.3) years, with slightly shorter disease duration in SPG7. SPRS was numerically higher in SPG11 as compared to SPG4 and SPG7.

### Physical activity

Analysis of IPAQ revealed that average patient-reported time spent sitting was highest in SPG11, albeit without significant differences to other genotypes (Fig. 1b-c). We observed a moderate correlation between SPRS and average daily time spent sitting (Spearman’s ρ = 0.53, *p* < 0.01, Fig. 1d). Higher SPRS was generally accompanied by a lower level of physical activity (Spearman’s ρ = -0.55, *p* < 0.001, Fig. 1e). Notably, some individuals with severe SPRS maintained a high degree of self-reported physical activity.

### Nutritional analysis

**Fig. 2:**
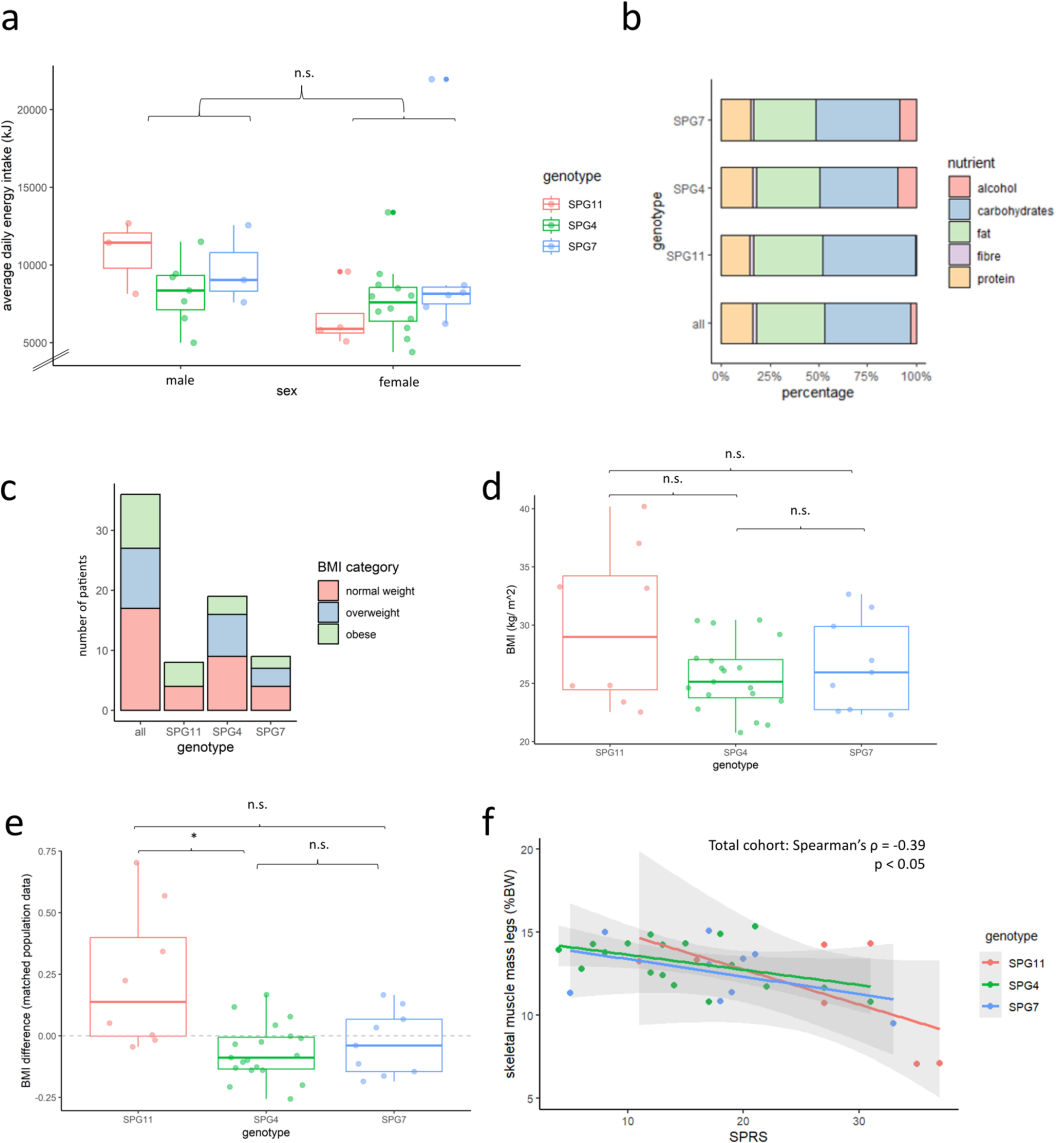
Baseline nutritional and body composition analysis. (**a**) Average daily energy value in kJ obtained through solids and fluids according to food diary. (**b**) Comparison of diet composition regarding macro nutrients, proportions in % of average energy intake per day. (**c**) Distribution of WHO-BMI-categories for different genotypes (normal weight: BMI ≤ 25 kg/ m^2^, overweight: 25–30 kg/ m^2^, obese: >30 kg/m^2^). (**d**) BMI at baseline for different genotypes. (**e**) BMI difference between the present study cohort and population data, matched by gender and age (Nationale Verzehrstudie II). (**f**) SPRS correlated significantly with skeletal muscle mass of the legs (total cohort Spearman’s ρ = -0.39, *p* < 0.05). *BMI* body mass index, *WHO* World Health Organization, *FMI* fat mass index, *n.s*. not significant. * *p* < 0.05 according to Kruskall-Wallis-Test followed by Dunn’s post-hoc test

Concerning average daily energy intake, there were no significant differences between sexes and genotypes (Fig. 2a, supplementary table T2). Moreover, a similar diet composition was observed across genotypes (Fig. 2b): Carbohydrates made up the largest proportion of average daily energy intake (44.1 ± 11.2%), followed by fat (34.7 ± 8.3%), protein (16.2 ± 3.6%), and alcohol (7.9 ± 11.5%). Dietary fiber made up the smallest proportion (2.0 ± 0.8%; 21.7 ± 15.6 g/d). The average intake of both protein and dietary fiber (in g/ d) correlated weakly with SPRS (protein: Spearman’s ρ = -0.35, *p* < 0.05; fiber: Spearman’s ρ = -0.42, *p* < 0.05).

### Anthropometry and body composition

According to the WHO criteria of obesity (Yumuk et al. 2015), 27.8% (10 patients) of the total cohort were overweight (BMI 25–29.9 kg/m^2^) and 25% (9 patients) were obese (BMI > 30 kg/ m^2^, Fig. 2c). Compared to SPG4 and SPG7, SPG11 showed numerically higher BMI values (Fig. 2d). The highest single BMI value (40.2 kg/m^2^) was present in the SPG11 sub-cohort.

In order to investigate BMI alterations in SPG11 further, we matched BMI values of our study cohort to German population data derived from reports on the German national nutrition study II (Nationale Verzehrsstudie II) (Max-Rubner-Institut, 2008a) by age and sex. Here, our SPG11 cohort showed a mean deviation of + 22.9% (median: 13.8%, SD: 26.7%). In contrast, SPG4 and SPG7 BMI distribution appears similar to the general German population (SPG4: -6.5 ± 10.9%, SPG7: -2.8 ± 12.5%; Fig. 2e). We calculated fat-mass-index (FMI = body fat (kg) / height (m)^2^) in order to normalize body fat percentage for height (VanItallie et al. 1990). There was a trend for higher mean FMI in SPG11 as compared to SPG4 and SPG7 (Table 2).

**Table 2:** Anthropometry and body composition at baseline for the total cohort and specific genotypes (Mean +/- SD, both sexes) *FMI* fat mass index; %*BW* percentage of body weight; %TBW percentage of total body water

BMI (kg/ m²)	Total cohort	SPG11	SPG4	SPG7
	26.8 ± 4.5	29.9 ± 6.4	25.6 ± 2.9	26.6 ± 3.7
Body fat (%BW)	33.2 ± 8.1	36.7 ± 12.1	31.5 ± 5.8	34.1 ± 7.4
FMI (kg/ m²)	9.0 ± 3.6	11.5 ± 6.3	8.1 ± 1.8	9.1 ± 2.4
skeletal muscle mass (%BW)	30.7 ± 5.5	29.3 ± 6.5	32.0 ± 4.0	29.3 ± 6.5
extracellular water (%TBW)	22.1 ± 2.1	20.3 ± 2.3	22.7 ± 1.8	22.3 ± 1.7

SPRS correlated negatively with skeletal muscle mass of the legs for the entire cohort (Spearman’s ρ = -0.39, *p* < 0.05, Fig. 2f). Extracellular water levels (% BW) determined by SMF-BIA were not significantly different between genotypes or sexes (supplemental figure F1). For SPG11 only, SPRS correlated with body fat percentage (SPG11: Spearman’s ρ = 0.76, *p* < 0.05; SPG4: Spearman’s ρ = 0.13; SPG7: Spearman’s ρ = 0.52, *p* = 0.15; see supp. Fig. F2a). Accordingly, in SPG11 longer disease duration was associated with higher body fat percentage (Spearman’s ρ = 0.67, *p* = 0.10) as well as higher FMI (Spearman’s ρ = 0.70, *p* = 0.08), and BMI (Spearman’s ρ = 0.56, *p* = 0.15). Similar correlations were found for SPG4, however weaker (supp. fig. F2a-b: body fat percentage and disease duration: Spearman’s ρ = -0.38, *p* = 0.12; BMI and disease duration: Spearman’s ρ = 0.44, *p* = 0.06).

In summary, the SPG11 cohort showed (non-significant) numerically higher BMI and FMI as compared to SPG4 and SPG7. Mean BMI of SPG11 exceeded matched population data significantly. Interestingly, the SPG11 sub-cohort consisted of just as much obese than normal weighted individuals. For SPG11, SPRS correlated with body fat percentage and BMI, and BMI correlated with disease duration. For the whole cohort, higher SPRS was associated with lower leg muscle mass.

### Metabolic blood profiling

**Fig. 3:**
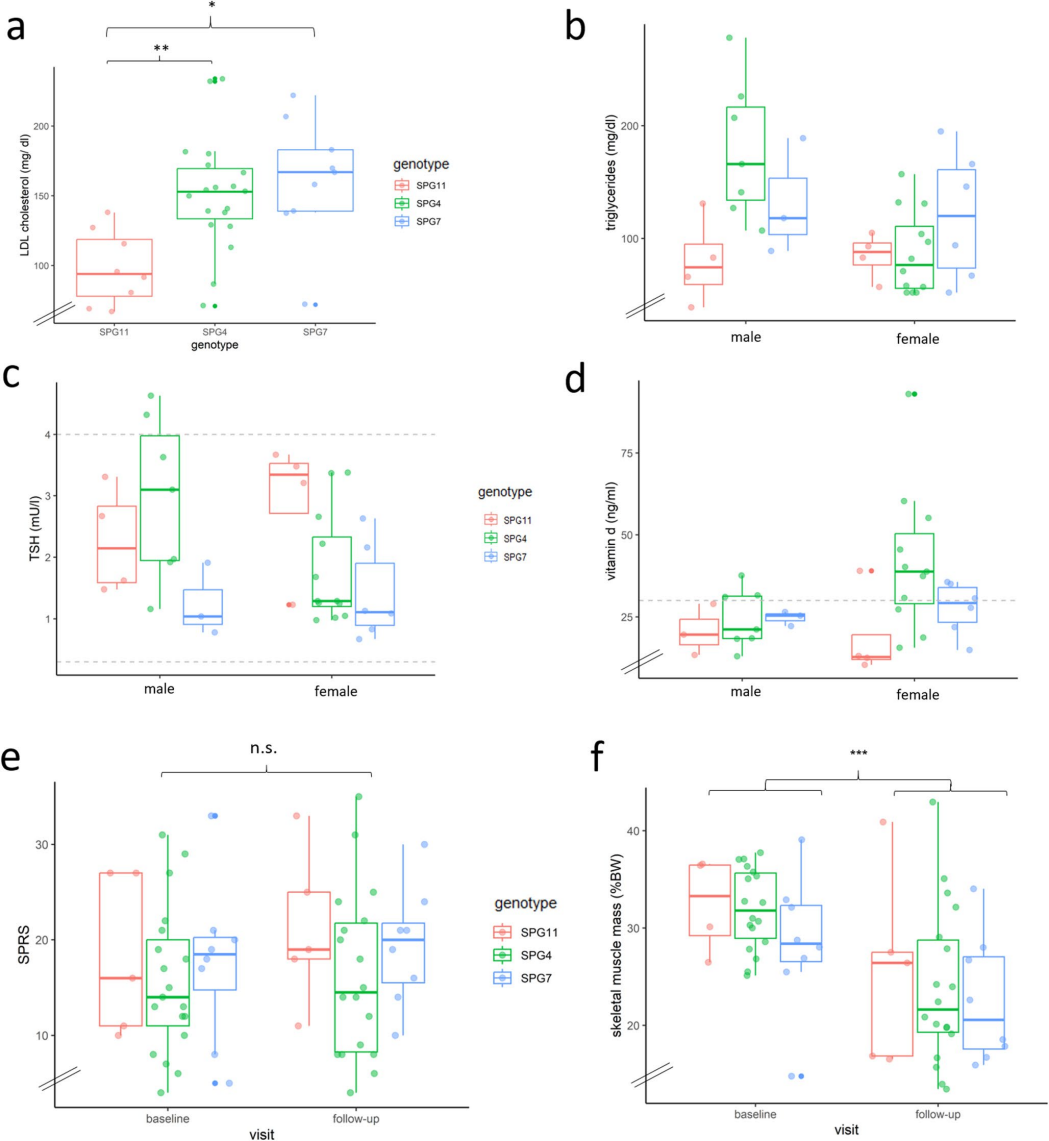
Metabolic status and longitudinal analysis. (**a**) LDL levels. (**b**) Triglyceride levels. (**c**) TSH levels (after exclusion of participants under substitution of levothyroxine; dotted lines represent reference range: 0.3–4.0 mU/l). (**d**) Vitamin D levels (dotted line represent cut-off for vitamin D deficiency: 30 ng/ml). (**e**) Disease severity as measured by SPRS for genotypes. Slight non-significant tendency of an increase in the 1-year-follow-up for the overall cohort mainly related to SPG11. (f) Longitudinal analysis of skeletal muscle mass (relative to total body weight). *LDL* low density lipoprotein, *TSH* thyroid stimulating hormone, *SPRS* Spastic Paraplegia Rating Scale, *TBW* total body weight. * *p* < 0.05; ** *p* < 0.01; *** *p* < 0.001; *n.s*. not significant according to Kruskall-Wallis-test followed by Dunn’s post-hoc test (a) or Wilcoxon signed-rank test (f)

Detailed results of the metabolic workup are presented in Table 3. No participant was under current therapy with statins or other medications directly acting on lipid metabolism. Significantly higher overall cholesterol and especially LDL levels were present in SPG4 and SPG7 as compared to SPG11 (*p* < 0.01 for SPG4 vs. SPG11, *p* < 0.05 for SPG7 vs. SPG11, Fig. 3a). Furthermore, we observed increased cholesterol levels in older vs. younger participants (mean 237.21 mg/dl in age > 35 years vs. 147.86 mg/dl in age < 35 years, *p* < 0.01; difference between means: 89,35 mg/dl, 60.4%). Accordingly, there was a weak positive correlation between blood cholesterol levels and age (Spearman’s ρ = 0,49; *p* < 0.01). There were no significant differences in triglyceride levels between genotypes (Fig. 3b).

**Table 3:** Metabolic characteristics at baseline for the total cohort and specific genotypes (Mean +/- SD, both sexes)

	Reference range	total cohort	SPG11	SPG4	SPG7
triglycerides(mg/dl)	50–200	113.1 ± 55.8	82.1 ± 26.9	120.9 ± 62.6	124.0 ± 49.6
cholesterol (mg/dl)	< 200	219.8 ± 57.0	154.4 ± 30.9	237.2 ± 47.3	241.3 ± 50.1
LDL		142.4 ± 44.2	98.3 ± 24.7	151.7 ± 39.3	161.8 ± 41.3
HDL		56.8 ± 17.3	43.6 ± 7.5	62.7 ± 19.5	56.1 ± 11.1
cortisol (ng/ml)		137.4 ± 50.2	135.4 ± 57.9	136.1 ± 56.1	142.2 ± 20.7
ACTH (pg/ml)	7.2–63.3	22.6 ± 9.5	30.0 ± 6.3	20.6 ± 9.6	17.9 ± 7.5
vitamin D (ng/ml)	30.0–70.0	29.7 ± 16.2	19.6 ± 9.9	35.2 ± 19.1	26.7 ± 6.2
TSH (mU/ l)	0.2-4.0	2.1 ± 1.1	2.6 ± 0.9	2.2 ± 1.2	1.4 ± 0.7
T3 (pmol/l)	3.2–7.2	6.3 ± 1.0	6.5 ± 1.0	6.2 ± 1.0	6.2 ± 0.6

Two participants (both male SPG4) met diagnostic criteria for diabetes, showing a HbA1c ≥ 6.5%. Four patients had a prediabetic metabolism according to HbA1c levels (≥ 5.7%). A comorbidity of arterial hypertension was present in 10 patients (SPG4 and SPG7, no SPG11).

SPG7 showed the lowest TSH serum levels, while SPG11 showed the highest levels (*p* < 0.05, Fig. 3c). Two participants showed TSH serum levels > 4.0 mU/l (both male SPG4). Triiodothyronine, thyroxine, cortisol and ACTH blood levels of the total cohort ranged within normal limits, without differences between genotypes and sexes.

Nine participants reported vitamin D supplementation (*n* = 6 SPG4, *n* = 2 SPG11, *n* = 1 SPG7; *n* = 8 female; *n* = 1 male). Nevertheless, mean vitamin D levels of the entire cohort were below the recommended value of 30 ng/ml, without significant genotype-specific differences (Fig. 3d). Lower vitamin D levels were associated with higher BMI (Spearman’s ρ = -0,56; *p* < 0.001), and FMI (Spearman’s ρ = -0,39; *p* < 0.05).

Taken together, we found significantly higher cholesterol levels for SPG4 and SPG7 compared to SPG11, as well as elder compared to younger participants. A substantial fraction of this HSP cohort failed to meet recommended vitamin D levels, especially female SPG11.

### Dietary recommendations and longitudinal analysis

The 1-year follow-up visit was conducted after 12.3 ± 1.5 (mean ± SD) months, with 4 patients lost to follow-up (mainly due to restrictions from the COVID-19 pandemic). SPRS scores worsened slightly compared to baseline, mainly driven by SPG11 (total cohort: 16.6 ± 7.7 points at baseline vs. 17.9 ± 7.9 points in follow-up; difference 1.3 points. SPG11: 18.2 ± 7.5 vs. 21.2 ± 7.4; difference 3.0; Fig. 3e).

Comparing baseline and follow-up in the overall cohort, most variables remained constant (average daily energy value, macro nutrients, BMI, body fat percentage, metabolic characteristics; supplemental fig. F3a-h; supplemental Fig. 4a-c). Notably, there was a significant decrease in skeletal muscle mass (%BW; difference between means total cohort: 7.2%, *p* < 0.001; Fig. 3f). This difference was present in both sexes and there was no pattern of genotype (%BW decrease in females: 9.4%, *p* < 0.01; males: 3.9%, *p* < 0.05; suppl. Figure 5a). There were no changes in the absolute amount of skeletal muscle mass or total body weight (suppl. Figure 5b-c).

Taken together, after 1 year of follow-up, relative skeletal muscle mass decreased along with increased SPRS, but there were no overall changes of nutrition or metabolic blood parameters.

### Obese and normal-weight SPG11

The SPG11 sub-cohort included an equal number of normal weight and obese participants according to WHO criteria (Yumuk et al. 2015) (see Table 4). Among these, obese SPG11 participants showed tendentially longer disease duration, higher SPRS, lower skeletal muscle mass (%BW; means: 25.07 vs. 32.42%), and higher TSH levels. Missing data points for IPAQ precluded a further analysis of physical activity in obese SPG11 participants. Interestingly, obese SPG11 participants also tended to report lower daily energy intake compared to normal-weight individuals.

**Table 4:** subgroup analysis of normal weight vs. overweight SPG11 (mean values) n number of participants

	*n*	BMI	Disease duration (years)	SPRS	TSH	Energy intake (kJ/ d)	Mean difference from population data: energy intake	Mean difference from population data: fat intake
normal-weight	4	23.9	13.75	11.0	1.89	2162.25	-10.13%	+ 1.29%
obese	4	35.9	18.75	28.25	3.28	1791.33	-22.14%	-17.67%

## Discussion

In this prospective longitudinal pilot study in HSP, we assessed the effects of a single nutritional counseling intervention on body composition, nutrition, and metabolism over a period of one year. The study cohort included the three most common genotypes of HSP with pure and complicated phenotypes (Blackstone 2018; de Souza et al. 2017; Fink 2013; Klebe et al. 2015). Therefore, the present cohort is representative for a European HSP cohort (de Bot et al. 2013; de Souza et al. 2017; Erfanian Omidvar et al. 2021; Schüle et al. 2016).

The one-year follow-up period was selected as a balance between the slow progression of HSP and the relatively short-term effects of dietary recommendations. The frequency of dietary counseling used in our study falls significantly below the usual frequency of nutritional counseling (Blumenthal et al. 2021; Deckers et al. 2024; Mitchell et al. 2017; Wong et al. 2022). One year after nutritional counseling, we observed no significant changes in nutrition, metabolic blood profiling, body composition nor anthropometry. We therefore conclude that a single 60-minute session of nutritional counseling is insufficient to induce measurable effects. It is likely that interventions of greater intensity and/or higher frequency are required to achieve clinically meaningful outcomes in future studies.

Given the modest increase in SPRS scores over the course of one year, longer follow-up periods should be implemented. A previous longitudinal long-term study in HSP showed that relevant disease progression (based on clinician-reported and gait performance outcomes) was only detected after 3 years and 4.5 years of follow-up which reflects the yet unresolved need for objective outcome parameters in HSP (Cubillos Arcila et al. 2023).

We observed no genotype-specific alterations in SPG4 and SPG7 concerning body composition or metabolic blood markers. The distribution of BMI and nutrition within these genotypes corresponded to the normal German population. Notably, metabolic syndrome was absent throughout the total cohort. Significantly increased cholesterol and LDL levels in SPG4 and SPG7 can be interpreted as an age effect, which is well known for the general population (Bertolotti et al. 2014; Kreisberg and Kasim 1987). Most participants exhibited vitamin D levels below the recommended range. The general association of low vitamin D levels and high BMI was also observed in the present HSP cohort (Earthman et al. 2012; Saneei et al. 2013).

Nevertheless, our analysis provides valuable insights into relations between body composition and physical activity in different types of HSP. SPRS correlated significantly with lower skeletal muscle mass of the legs and lower SPRS was associated with higher average daily protein intake. This may highlight the importance of sufficient protein intake to prevent muscle wasting/ sarcopenia, thus preserving functional status (Calvani et al. 2023; Cruz-Jentoft and Sayer 2019; Nascimento et al. 2019). Our data confirm that more severely affected patients as measured by SPRS also show a lower level of physical activity. Based on our data, an intervention combining weight training and a high-protein diet may have disease-modifying potential.

The macro-nutrient diet composition recorded in our HSP cohort was similar throughout genotypes, and overall comparable to the general German population (Max-Rubner-Institut, 2008b). Disease severity was associated with less dietary fiber and protein intake. This finding supports the possible disease modifying potential through nutritional intervention mentioned above. Nevertheless, in the absence of a sensitive biomarker for disease progression, it currently seems not feasible to assess the impact of dietary/ physical intervention on disease severity and progression in HSP in general.

Comparing BMI, FMI and body fat percentage between genotypes, we observed numerically higher values for SPG11. Because of the limited number of SPG11 individuals with only 4 fulfilling the WHO criteria of obesity, this observation must be interpreted with caution. In addition, our interpretation may be limited by the inclusion of identical SPG11 twins with extreme BMI and body fat percentage, which may be caused by additional (genetic) causes. Nevertheless, previous single-center studies reported similar observations (Cardozo-Hernández et al. 2020; Regensburger et al. 2022a, b). Further research is needed to probe the rigidity of a probable “SPG11 specific phenotype”.

The correlations between body fat percentage/ BMI with disease duration/ SPRS may underline a potential tendency for obesity in SPG11 evolving with increasing disease duration and severity. We relate the non-significant testing to the small number of recruitment typical for investigating rare diseases. Of note, disease duration and BMI/ body fat percentage also correlated in the SPG4 cohort (albeit at a weaker degree). Ideally, the relation between anthropometry and disease course would have to be investigated in prospective multi-center studies with larger sub-cohorts across different disease stages in different genotypes. Investigation of anthropometry and metabolism should be complemented by the assessment of nutrition and physical activity.

The analysis of genotype-specific anthropometry in relation to nutrition and metabolic markers in SPG11 contains particularly noteworthy findings. The SPG11 sub-cohort consisted of equally normal-weight and obese individuals, showing a BMI well over 30 (each *n* = 4). Surprisingly, obese SPG11 showed a tendency for lower energy intake compared to normal-weighted individuals of the same genotype (at baseline, as well as the follow-up visit. Without significant changes between the two timepoints). Again, these findings have to be interpreted with caution, since nutritional analysis was based on self-assessment and data might be influenced by various biases. Still, generally lower energy intake in obese individuals wouldargue against overfeeding or hyperphagia as a potential cause, as well as the previously suggested leptin resistance in SPG11 (Regensburger et al. 2022a, b). Considering this, determining resting energy expenditure seems essential for future studies.

This assumption supports the hypothesis that the hypothalamic atrophy present in SPG11 (Cardozo-Hernández et al. 2020; Regensburger et al. 2022a, b) may be directly linked to obesity through neurodegenerative metabolic alterations. Physiologically, the hypothalamus controls energy expenditure and satiation via endocrine signaling pathways including the hypothalamus-pituitary axis and the sympathetic nervous system (Brüning and Fenselau 2023; Fong et al. 2023). SPG11 is known to be expressed within the hypothalamus, but also in thyroid, adipose and adrenal gland tissue (according to the Human Protein Atlas (Uhlén et al. 2015). Though there were no overall metabolic alterations in the total SPG11 cohort, obese SPG11 showed numerically higher mean TSH (without fulfilling the criteria of manifest hypothyreosis). The lymphedema of the lower extremities previously reported in SPG11 (Cardozo-Hernández et al. 2020; Regensburger et al. 2022a, b) are also found in hypothyroidism and may support shared pathophysiological mechanisms. The link between obesity and metabolism in HSP remains speculation until further studies shed light on the validity of such assumptions.

In fact, the association between hypothalamic atrophy and obesity is well established in the pathophysiology of acquired hypothalamic obesity, which occurs in hypothalamic damage of various causes (e.g. genetic variants in Prader-Willi syndrome, brain tumors, or iatrogenic) (Argente et al. 2025). Notably, the metabolic findings in acquired hypothalamic atrophy appear similar to those observed in our SPG11 sub-cohort: affected patients typically show higher BMI and lower resting energy expenditure together with decreased physical activity (Harz et al. 2003; Shaikh et al. 2008). There are also reports that suggest lower energy intake (mainly driven by reduced fat (Harz et al. 2003). Therefore, similar pathophysiological mechanisms may be at play in both conditions.

The results of our study are in line with previous findings of an increased BMI in SPG11-HSP compared to normal population or other neurodegenerative diseases (Cardozo-Hernandez et al., 2020, Regensburger et al. 2022a, b). In the future, the hypothesis of a potential genotype-specific obese phenotype in SPG11 should be tested in a larger, multi-center, genetically stratified study cohort, ideally within a prospective study design. It would be imperative to assess resting energy expenditure along with physical activity. If the hypotheses cautiously derived from our purely exploratory data are confirmed, investigating the underlying causes of obesity may reveal relevant therapeutic opportunities.

Lastly, pathophysiological alterations in lipid metabolism in SPG11 represents another interesting avenue for potential links between metabolism and neurodegeneration in HSP. Spastacsin, encoded by SPG11, is essential for lysosomal function. Although the pathophysiological mechanisms of mutated spastacsin that ultimately lead to neurodegeneration are not yet fully elucidated, mitochondrial dysfunction and lipid accumulation were found to play an important role (Awuah et al. 2024; Güner et al. 2021; Hehr et al. 2007). In a recent case report, SPG11 was found to mimic gangliosidosis, with lipid accumulations observed within enteric neurons (Lopergolo et al. 2022).

The present study is limited by the small sample size that was included for each genotype, which is a common limitation in rare disease studies. For this reason, we did not perform correction for multiple testing which is why the present findings have to be interpreted with caution and have to be regarded as explorative.

## Conclusion

In conclusion, our study provides valuable insights into metabolism in HSP. Within the obese SPG11 subcohort, we explored potential indications of metabolic alterations similar to acquired hypothalamic obesity, suggesting overlapping pathomechanisms, potentially caused by hypothalamic hypotrophy described previously for this genotype.

Across all subgroups, affected individuals showed reduced physical activity and lower skeletal muscle mass after one year, both of which are known to negatively influence fat mass regulation, independent of dietary intake. These findings support the notion that obesity in HSP may arise from a combination of reduced mobility, disease-related muscle loss, and metabolic alterations. This finding seems to be especially true for SPG11-related HSP.

Alongside targeted nutritional support, a protein-rich diet and structured strength training may help to preserve or improve muscle mass, which could in turn positively influence the metabolic profile and potentially the disease course. Future studies should further explore the relationship between physical activity, hypothalamic dysfunction, body composition, and resting energy expenditure across different HSP genotypes.

## Supplementary Information

Below is the link to the electronic supplementary material.

Supplementary Material 1

## Data Availability

The data that support the findings of this study are available from the corresponding author upon reasonable request.
