# Supplementary material for: Beyond mobility: A prospective study on diet and metabolism in hereditary spastic paraplegia
Source: Metab Brain Dis. 2026 Mar 3;41(1):45. doi: 10.1007/s11011-026-01815-x (PMC12956933; doi:10.1007/s11011-026-01815-x)
Supplement: Supplementary file 1 — Supplementary Material 1 [file 11011_2026_1815_MOESM1_ESM.docx]

***Beyond Mobility: A prospective interventional study on diet and metabolism in Hereditary Spastic Paraplegia***

Christina Erhardt^1^, Imke T. Spatz^1^, Hans J. Herrmann^2^, Zacharias Kohl^1,3^, Yurdagül Zopf^2^, Heiko Gassner^1,4^, Jürgen Winkler^1^, Martin Regensburger^1,5^

1. Department of Molecular Neurology, Friedrich-Alexander-Universität Erlangen-Nürnberg, Erlangen, Germany
2. Hector-Center for Nutrition, Exercise and Sports, Department of Medicine 1, Friedrich-Alexander-Universität Erlangen-Nürnberg, Erlangen, Germany
3. Department of Neurology, University Hospital Regensburg, Regensburg, Germany
4. Fraunhofer Institute for Integrated Circuits IIS, Erlangen, Germany
5. Deutsches Zentrum Immuntherapie (DZI), Erlangen, Germany

**Corresponding author:**

Martin Regensburger, MD, Department of Molecular Neurology, Schwabachanlage 6, 91054 Erlangen, Germany; Email: [martin.regensburger@uk-erlangen.de](mailto:martin.regensburger@uk-erlangen.de)

## Supplementary Tables

### **Supplementary Table T1:** baseline characteristics and assessment of physical activity at baseline (genotypes + sexes)

Mean +/- SD – all sexes/ male/ female

|  | **HSP total** | | **SPG11** | | **SPG4** | | **SPG7** | |
| --- | --- | --- | --- | --- | --- | --- | --- | --- |
| n | 36 | | 8 (22.2%) | | 19 (52.8%) | | 9 (25.0%) | |
| female | 22 | | 4 | | 12 | | 6 | |
| male | 14 | | 4 | | 7 | | 3 | |
| length of follow-up (months) | 12.3 ± 1.5 | | 12.6 ± 1.8 | | 12.6 ± 1.3 | | 11.5 ± 1.4 | |
| n of follow-up | 32 | | 5 | | 19 | | 8 | |
| age (years) | 48.3 ± 13.8 | | 25.9 ± 7.6 | | 54.4 ± 6.8 | | 55.3 ± 6.1 | |
|  | 47.0  ± 14.0 | 49.1  ± 13.6 | 28.8  ± 9.8 | 23.0  ± 2.0 | 53.4  ± 7.9 | 55.0  ± 6.0 | 56.3  ± 3.1 | 54.8  ± 7.1 |
| disease duration (years) | 15.6 ± 11.3 | | 16.3 ± 8.3 | | 16.9 ± 12.4 | | 12.4 ± 10.5 | |
|  | 19.3  ± 13.3 | 13.3  ± 9.1 | 14.5  ± 8.4 | 18.0  ± 7.8 | 26.9  ± 13.3 | 11.1  ± 6.9 | 8.0  ± 4.9 | 14.7  ± 11.8 |
| SPRS  (total sum score, points) | 18.1 ± 8.8 | | 24.3 ± 9.9 | | 15.7 ± 7.5 | | 17.6 ± 7.5 | |
|  | 17.4  ± 7.3 | 18.5  ± 9.6 | 21.0  ± 8.4 | 27.5  ± 10.2 | 16.7  ± 6.9 | 15.1  ± 7.8 | 14.0  ± 4.2 | 19.3  ± 8.1 |
| Time spent sitting (min/ day) | 396.3  ± 187.6 | | 478.6  ±157.6 | | 387.9  ± 158.5 | | 359.0  ± 240.4 | |
| Time spent with physical activity (min/day) | 159.9 ± 171.4 | | 151.0 ± 203.7 | | 175.5 ± 175.3 | | 133.0 ± 135.3 | |
| Total physical activity (MET-min/ week) | 4827.3 ± 5621.8 | | 5591.0 ± 7882.3 | | 4839.4 ± 4743.3 | | 4292.6 ± 5618.8 | |

**Supplementary Table T2:** nutritional analysis at baseline for genotypes and sexes

| Average daily energy intake (kJ) | 8531.1 ± 3174.9 | | 8390.6 ± 2772.5 | | 7903.4 ± 2144.2 | | 9965.7 ± 4549.8 | |
| --- | --- | --- | --- | --- | --- | --- | --- | --- |
|  | 9172.1 ± 2245.7 | 8152.3 ± 3560.1 | 10760.3 ± 2034.1 | 6613.2 ± 1746.0 | 8251.6 ± 1951.2 | 7700.2 ± 2224.0 | 9732.0 ± 2085.6 | 10082.5 ± 5369.9 |
| Average daily protein intake (% average daily energy intake) | 16.2 ± 3.6 | | 14.4 ±1.7 | | 17.1 ± 3.9 | | 15.8 ± 3.7 | |
| Average daily protein intake (g) | 78.9 ± 24.6 | | 74.5 ± 24.2 | | 77.7 ± 25.6 | | 85.2 ± 21.2 | |
|  | 86.6  ± 14.8 | 73.9  ± 28.1 | 95.6  ± 5.2 | 53.3  ± 15.8 | 78.9  ± 16.9 | 77.0  ± 29.6 | 92.6  ± 5.7 | 81.4  ± 24.8 |
| Average daily protein intake per TBW (g/kg) | 1.06 ± 0.4 | | 0.9 ± 0.3 | | 1.0 ± 0.4 | | 1.2 ± 0.4 | |
|  | 1.0  ± 0.3 | 1.1  ± 0.5 | 1.1  ± 0.2 | 0.8  ± 0.4 | 0.9  ± 0.3 | 1.2  ± 0.4 | 1.1  ± 0.1 | 1.2  ± 0.5 |
| Average daily fat intake (% average daily energy intake) | 34.7 ± 8.3 | | 35.6 ± 10.6 | | 34.8 ± 7.0 | | 33.7 ± 8.9 | |
| Average daily carbohydrate intake (% average daily energy intake) | 44.1 ± 11.2 | | 47.9 ± 9.9 | | 42.1 ± 10.9 | | 45.5 ± 11.6 | |
| Average daily fiber intake (% average daily energy intake) | 2.0 ± 0.8 | | 2.0 ± 0.8 | | 2.0 ± 0.7 | | 2.0 ± 1.1 | |
| Average daily fiber intake (g/ d) | 21.7 ± 15.6 | | 21.9 ± 11.5 | | 19.1 ± 9.9 | | 27.8 ± 24.7 | |
| Average daily alcohol intake (% average daily energy intake) | 2.9 ± 3.8 | | 0.25 ± 0.4 | | 4.2 ± 4.0 | | 2.4 ± 3.8 | |
| Average daily alcohol intake (g) | 7.9 ± 11.5 | | 0.8 ± 1.3 | | 10.4 ±10.2 | | 9.1 ± 15.6 | |

### **Supplementary Table T3:** metabolic characteristics at baseline

Mean +/- SD – all sexes/ male/ female

|  | **HSP total** | | **SPG11** | | **SPG4** | | **SPG7** | |
| --- | --- | --- | --- | --- | --- | --- | --- | --- |
| leptin (ng/ml) | 23.01 ± 26.7 | | 46.1 ± 46.7 | | 14.0 ± 8.1 | | 21.4 ± 11.1 | |
|  | 11.7  ± 7.0 | 30.2  ± 31.6 | 13.75  ± 7.7 | 78.5  ± 46.9 | 9.2  ± 2.8 | 16.8  ± 8.9 | 14.7 ±10.2 | 24.8 ±10.0 |
| triglycerides  (50-200 mg/dl) | 113.1 ± 55.8 | | 82.1 ± 26.9 | | 120.9 ± 62.6 | | 124.0 ± 49.6 | |
|  | 140.5  ± 64.0 | 95.6  ± 41.4 | 79.7  ± 3.5 | 84.5  ± 17.7 | 178.9  ± 56.4 | 87.1 ± 35.3 | 136.0  ± 43.0 | 120.0 ± 52.5 |
| cholesterol  (<200 mg/dl) | 219.8 ± 57.0 | | 154.4 ± 30.9 | | 237.2 ± 47.3 | | 241.3 ± 50.1 | |
|  | 224.1  ± 56.8 | 217.1 ± 56.9 | 165.7  ± 30.8 | 143.0  ± 26.5 | 256.6  ± 53.3 | 225.9  ± 39.1 | 226.3  ± 11.9 | 248.8  ± 59.4 |
| LDL | 142.4 ± 44.2 | | 98.3 ± 24.7 | | 151.7 ± 39.3 | | 161.8 ± 41.3 | |
|  | 151.8  ± 42.4 | 136.3 ± 44.3 | 109.5  ± 23.6 | 87.0  ± 20.3 | 174.8  ± 40.6 | 138.2  ± 31.5 | 154.3  ± 12.1 | 165.5  ± 49.4 |
| HDL | 56.8 ± 17.3 | | 43.6 ± 7.5 | | 62.7 ± 19.5 | | 56.1 ± 11.1 | |
|  | 48.7  ± 12.1 | 62.0  ± 18.2 | 42.7  ± 5.9 | 44.5  ± 8.7 | 52.1  ± 15.3 | 68.9  ± 19.0 | 48.7  ± 3.7 | 59.8  ± 11.6 |
| HbA1c  (4.4 - 6.0%) | 5.4 ± 0.5 | | 5.2 ± 0.2 | | 5.5 ± 0.6 | | 5.5 ± 0.2 | |
|  | 5.6  ± 0.7 | 5.3  ± 0.2 | 5.2  ± 0.0 | 5.3  ± 0.3 | 5.9  ± 0.9 | 5.3  ± 0.2 | 5.5  ± 0.3 | 5.5  ± 0.2 |
| cortisol (ng/ml) | 137.4 ± 50.2 | | 135.4 ± 57.9 | | 136.1 ± 56.1 | | 142.2 ± 20.7 | |
|  | 150.2  ±39.9 | 129.3  ± 54.2 | 146.3  ± 40.9 | 124.5  ± 69.3 | 162.4  ± 42.8 | 120.7  ± 57.2 | 127.0  ± 7.3 | 149.8  ± 21.0 |
| ACTH  (7.2 - 63.3 pg/ml) | 22.1 ± 9.5 | | 30.0 ± 6.3 | | 20.6 ± 9.6 | | 17.9 ± 7.5 | |
|  | 26.4  ±10.1 | 19.3  ± 8.0 | 29.5  ± 7.2 | 30.6  ± 5.3 | 29.2  ± 8.3 | 15.6  ± 6.0 | 15.2  ± 10.0 | 19.0  ± 5.6 |
| vitamin D  (30.0 – 70.0 ng/ml) | 29.7 ± 16.2 | | 19.6 ± 9.9 | | 35.2 ± 19.1 | | 26.7 ± 6.2 | |
|  | 23.6  ± 7.1 | 33.5 ±18.9 | 20.7  ± 6.8 | 18.7  ± 11.7 | 24.5  ± 8.3 | 42.1  ± 20.8 | 24.7  ± 1.8 | 27.7  ± 7.3 |
| TSH (0.2-4.0 mU/ l) | 2.1 ±1.1 | | 2.6 ± 0.9 | | 2.2 ± 1.2 | | 1.4 ± 0.7 | |
|  | 2.4 ±1.2 | 2.0 ±1.0 | 2.9  ± 1.2 | 1.8  ± 0.9 | 2.1  ± 1.2 | 1.8  ± 0.9 | 1.2  ± 0.5 | 1.5  ± 0.7 |
| T3 (3.2-7.2 pmol/l) | 6.3 ±1.0 | | 6.5 ±1.0 | | 6.2 ± 1.0 | | 6.2 ± 0.6 | |
|  | 6.7  ± 1.1 | 5.9  ± 0.7 | 7.4  ± 0.6 | 5.6  ± 0.6 | 6.8  ± 1.4 | 5.8  ± 0.6 | 5.8  ± 0.2 | 6.4  ± 0.6 |

## Supplementary Figures

### Supplementary Fig. F1 **Anthropometry and body composition**. *a:* Body fat percentage (%BW) grouped by sex for each genotype (colors) shows significant difference for higher body fat in females irrespective of genotype (p<0.001 according to unpaired non-parametric Wilcoxon test. *% BW* percentage of total body weight. *b:* Skeletal muscle mass (%BW) grouped by sex for each genotype (colors) shows significantly altered skeletal muscle mass in male participants (*** p<0.001 according to unpaired non-parametric Wilcoxon test). *% TBW* percentage of total body weight. *c:* Extracellular water (%BW) grouped by sex for each genotype (colors) shows no significant differences (according to unpaired Wilcoxon non-parametric testing). %BW percentage of total body weight.


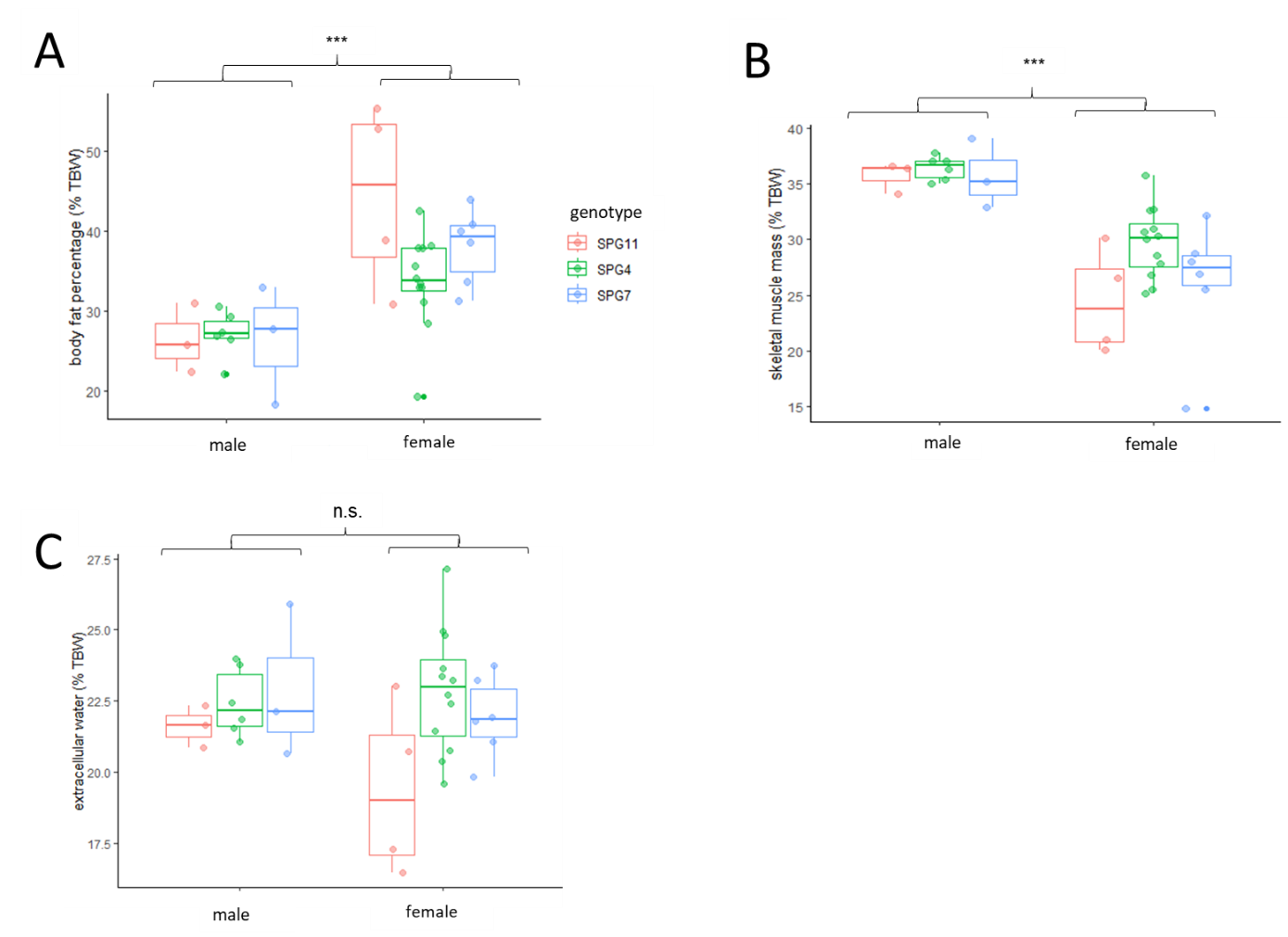


c

b

a

### Supplemental Fig. F2 **Correlations between anthropometry and body composition with disease duration and SPRS**. *a:* correlations between body fat percentage and SPRS. Strong, significant correlation for SPG11: Spearman’s ρ = 0.76, p < 0.05; no correlation for SPG4: Spearman’s ρ = 0.13; moderate, non-significant correlation for SPG7: Spearman’s ρ = 0.52, p = 0.1. *b:* correlations between BMI and disease duration. Moderate, non-significant correlation for SPG11: Spearman’s ρ =0.56, p = 0.15; weak, non-significant correlation for SPG4: Spearman’s ρ = 0.44, p =0.06.


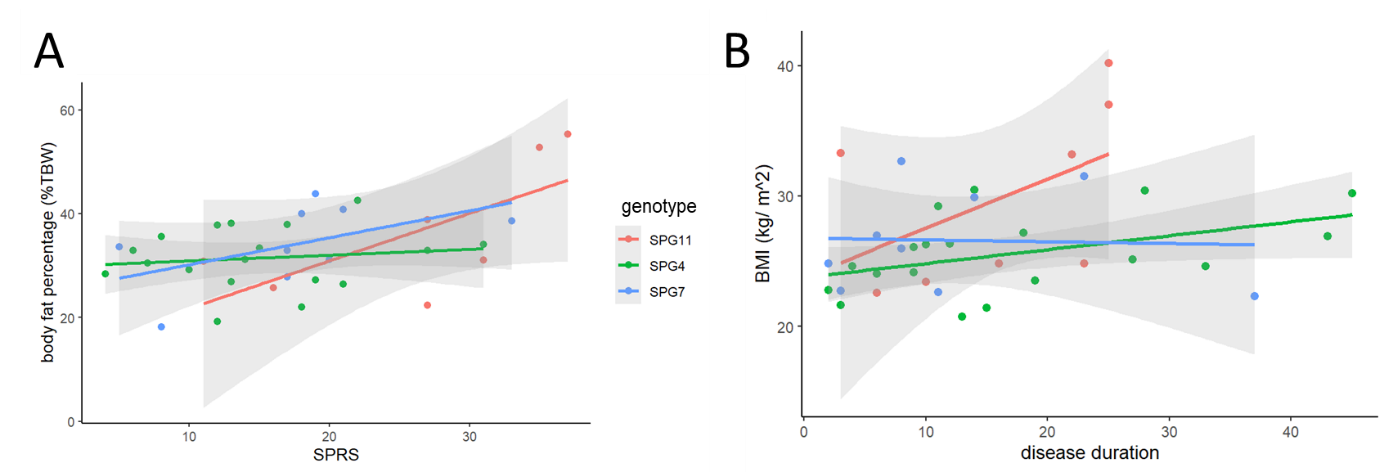


b

a

### Supplemental Fig. F3: **Longitudinal analysis of anthropometry, metabolism, body composition**. *a:* average daily energy intake in kJ in comparison of baseline and follow-up visit, grouped by genotype (colors). No significant longitudinal difference according to non-parametric Wilcoxon test (paired). *b*: average daily carbohydrate intake in percentage of average daily energy value (%EV) at baseline and follow-up, grouped by genotype. No significant difference according to non-parametric Wilcoxon test (paired). *d:* average daily protein intake in percentage of average daily energy value (%EV) at baseline and follow-up, grouped by genotype. No significant difference according to non-parametric Wilcoxon test (paired). *D:* average daily fat intake in percentage of average daily energy value (%EV) at baseline and follow-up, grouped by genotype. No significant difference according to non-parametric Wilcoxon test (paired). *e:* average daily alcohol intake in percentage of average daily energy value (%EV) at baseline and follow-up, grouped by genotype. No significant difference according to non-parametric Wilcoxon test (paired). *f*: body mass index (BMI) at baseline and follow-up, grouped by genotype. No significant difference according to non-parametric Wilcoxon test (paired). *g:* body fat percentage in percentage of total body weight at baseline and follow-up, grouped by genotype. No significant difference according to non-parametric Wilcoxon test (paired). *h:* extracellular water in percentage of total body weight at baseline and follow-up, grouped by genotype. No significant difference according to non-parametric Wilcoxon test (paired).


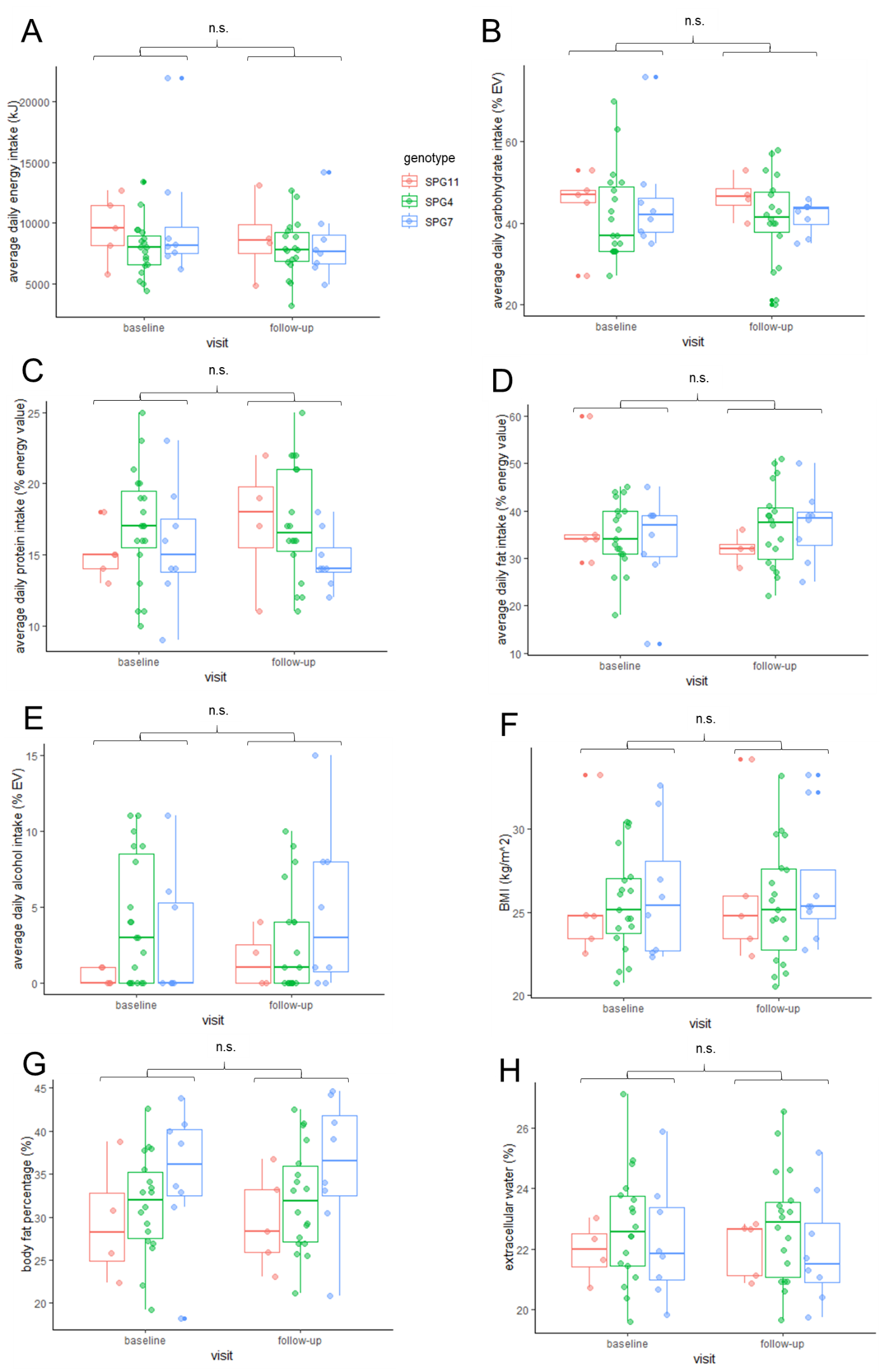


h

g

f

e

d

c

b

a

### Supplementary Fig. F4: **Longitudinal analysis of lipid metabolism parameters.** *A:* triglycerides in blood serum at baseline and follow-up, grouped by genotype. No significant difference according to non-parametric Wilcoxon test (paired). *B:* total cholesterol in blood serum at baseline and follow-up, grouped by genotype. No significant difference according to non-parametric Wilcoxon test (paired). *C:* LDL cholesterol in blood serum at baseline and follow-up, grouped by genotype. No significant difference according to non-parametric Wilcoxon test (paired).

b

a


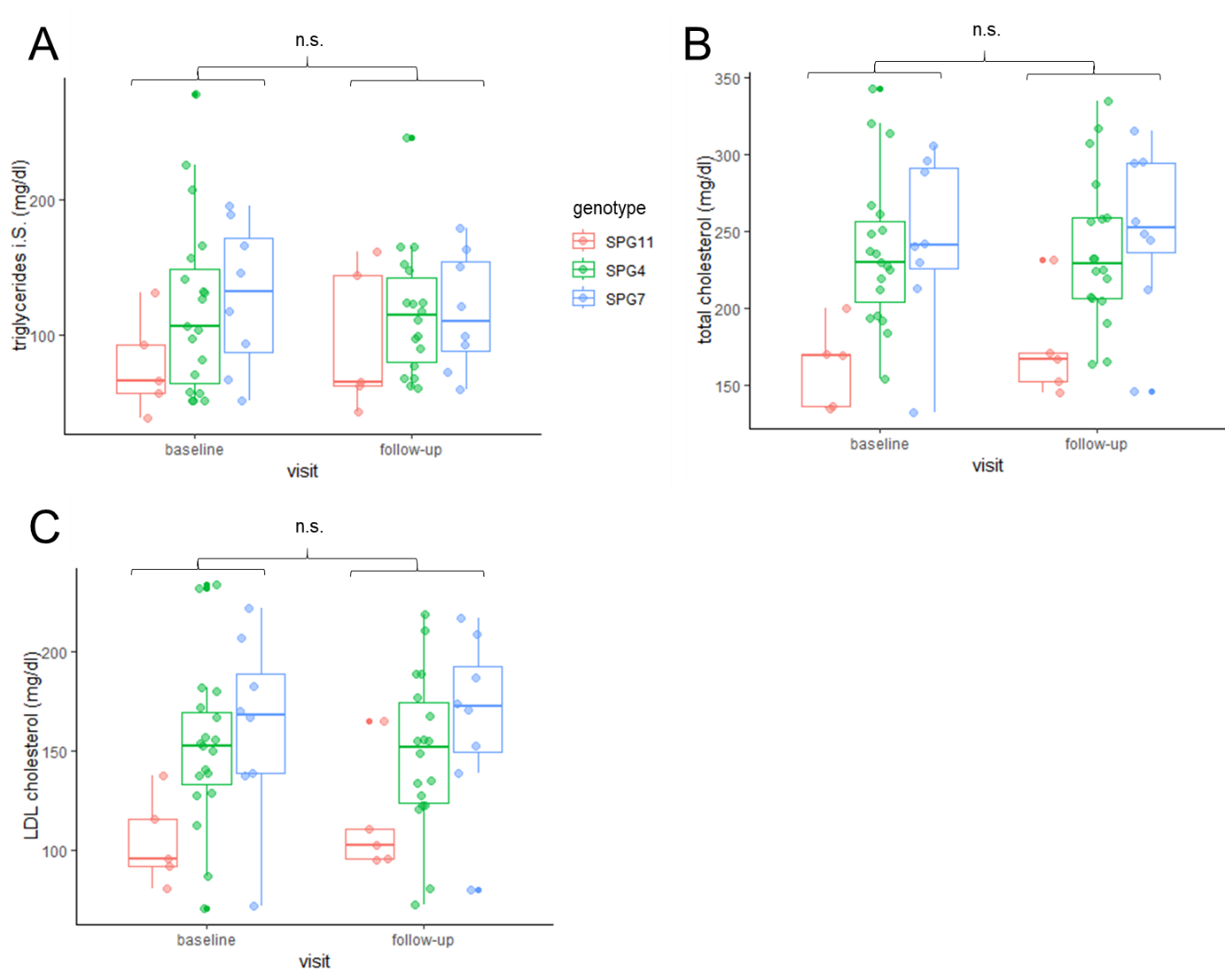


c

### Supplemental Fig. F5: **Longitudinal changes in skeletal muscle mass.** *A:* Skeletal muscle mass in percentage of total body weight at baseline and follow-up visit, grouped by sexes (colors). Significantly lower skeletal muscle mass especially in female participants at the follow-up visit (*** p<0.001, *p<0.05 according to paired, non-parametric Wilcoxon-test). *B:* absolute skeletal muscle mass in kg at baseline and follow-up visit, grouped by genotype (colors). No significant difference according to non-parametric Wilcoxon test (paired). *C:* total body weight in kg at baseline and follow-up visit, grouped by sexes (colors). No significant difference according to non-parametric Wilcoxon test (paired).


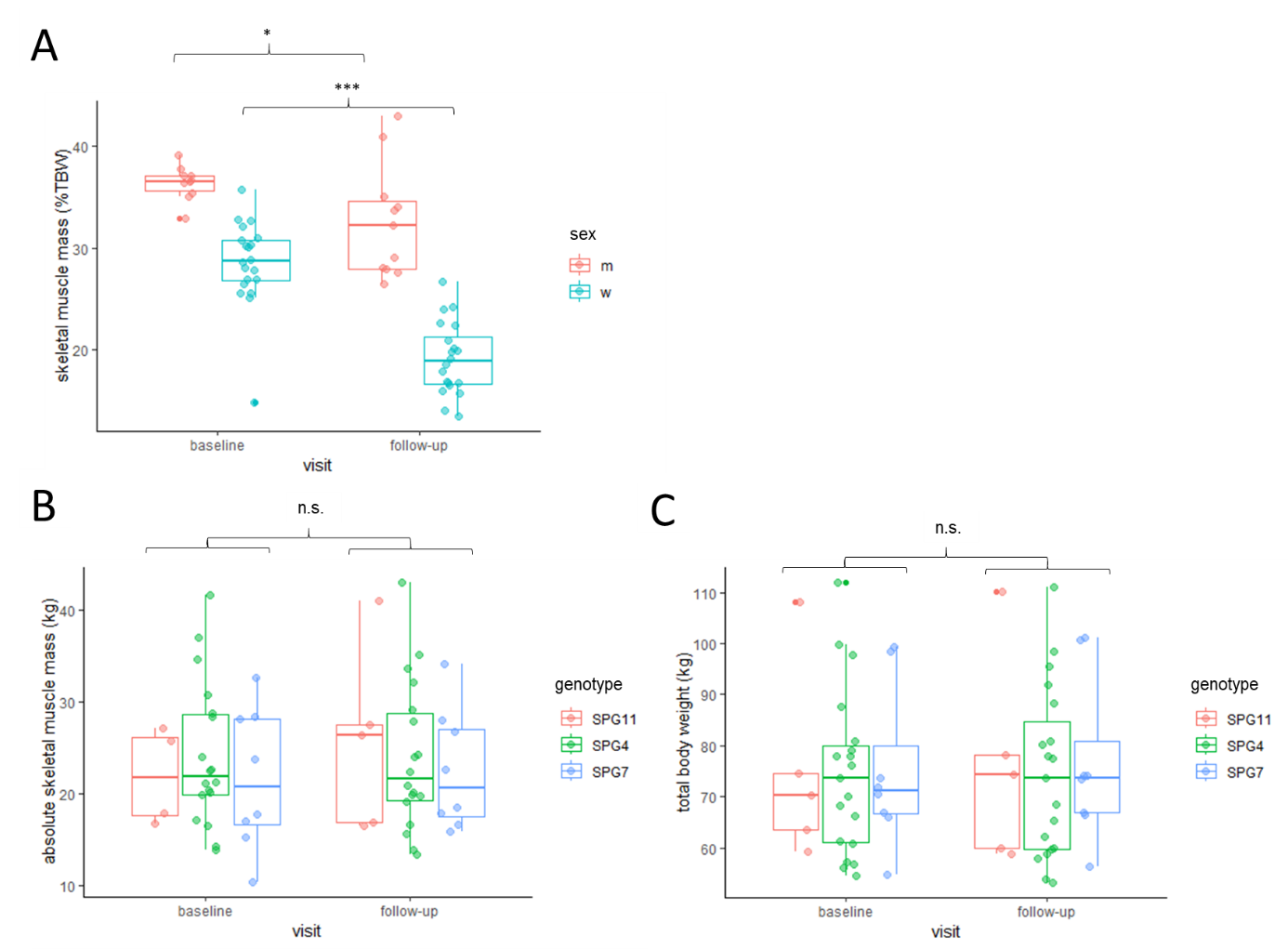


male

female

c

b

a
